# Microgravity versus Microgravity and Irradiation: Investigating the Change of Neuroendocrine-Immune System and the Antagonistic Effect of Traditional Chinese Medicine Formula

**DOI:** 10.1155/2020/2641324

**Published:** 2020-05-26

**Authors:** Haoru Zhu, Lin Zhang, Meng Qian, Tuo Shi, Fangxin Fan, Wenfei Li, Sitai Zhu, Ming Xie

**Affiliations:** ^1^School of Traditional Chinese Medicine, Bejing University of Chinese Medicine, Beijing 100029, China; ^2^School of Traditional Chinese Medicine, Liaoning University of Chinese Medicine, Shenyang, Liaoning 110847, China

## Abstract

During spaceflight, the homeostasis of the living body is threatened with cosmic environment including microgravity and irradiation. Traditional Chinese medicine could ameliorate the internal imbalance during spaceflight, but its mechanism is still unclear. In this article, we compared the difference of neuroendocrine-immune balance between simulated microgravity (S) and simulated microgravity and irradiation (SAI) environment. We also observed the antagonistic effect of SAI using a traditional Chinese medicine formula (TCMF). Wistar rats were, respectively, exposed under S using tail suspending and SAI using tail suspending and ^60^Co-gama irradiation exposure. The SAI rats were intervened with TCMF. The changes of hypothalamic–pituitary–adrenal (HPA) axis, splenic T-cell, celiac macrophages, and related cytokines were observed after 21 days. Compared with the normal group, the hyperfunction of HPA axis and celiac macrophages, as well as the hypofunction of splenic T-cells, was observed in both the S and SAI group. Compared with the S group, the levels of plasmatic corticotropin-releasing hormone (CRH), macrophage activity, and serous interleukin-6 (IL-6) in the SAI group were significantly reduced. The dysfunctional targets were mostly reversed in the TCMF group. Both S and SAI could lead to NEI imbalance. Irradiation could aggravate the negative feedback inhibition of HPA axis and macrophages caused by S. TCMF could ameliorate the NEI dysfunction caused by SAI.

## 1. Introduction

Since the last century, the exploration of space has been gradually unfolding. However, the physiological behavior of human beings is continuously threatened with extreme environmental factors in space, such as microgravity, irradiation, space noise, narrow space, and social loneliness, which has seriously affected the physical and mental health of astronauts and hindered the development of manned spaceflight partly [1].

Microgravity and irradiation are the most important factors among the complex space environment. It is reported that microgravity could affect multiple physiological systems including the cardio-cerebral-vascular system [2], nervous system [3], locomotor system [4], and immune system [5]. On the other hand, it is reported that space irradiation could lead to the dysfunction of the nervous system [6], endocrine system [7], and immune system [8]. Considering the difficulties of midcourse space experiment, such as high research cost and unsatisfied experimental space, the research on space extreme environment is generally simulated on the ground. However, most of the studies are currently focused on physiological changes caused by a single environmental factor like microgravity or irradiation, how the complexed extreme environment interferes with the human body is barely researched.

Since the immune-neuro-endocrine system was hypothesized by Besedovsky and Sorkin in 1977 [9], researchers have been proceeding a variety of studies on the internal mechanism of the neuroendocrine and immune systems. It has been confirmed that there exists a bidirectional mechanism between the neuroendocrine system and immune system, which build the neuro-endocrine-immune (NEI) system together via a synergistic effect and antagonism. The NEI system plays an important role in homeostasis and against external environmental aggression. It is reported that the NEI system could engender a series of changes to readapt the new external environment while facing the extreme space surroundings. Nevertheless, the research on how the NEI system changes under microgravity-irradiation environment is scarcely reported.

Traditional Chinese medicine (TCM) shows its advantage on systematically reconciling the physiological function of the human body. It is reported that traditional Chinese medicine formula (TCMF), especially which is used based on TCM theory, could effectively alleviate the physical impairment caused by microgravity, irradiation, and other space environments [10]. TCMF “Taikong Xieli Decoction” (TKXLD), which was formulated by our team, has been confirmed to modulate the immunity of rats under the condition of short-term microgravity combined with irradiation [11]. However, the immunomodulatory mechanism of TKXLD is still unclear.

Overall, the research on organism adaption in space is depending rapidly, yet a lot of deficiencies still remain, especially on the comprehensive consideration of the complex space environment, systematized exploration on multiple physiological systems in the human body, and profound study on the mechanism of TCMF. Hence, we reconstructed the rat model which suffered from microgravity and irradiation to observe the changes in the NEI system and the modulated the mechanism of TKXLD. This study might give experimental support for ascertaining the influence of space environment on a mammal and the antagonistic effect of TCMF.

## 2. Materials and Methods

### 2.1. Preparation of TKXLD

TKXLD consists of Ginseng Radix et Rhizoma (*PanaxginsengC.A.Mey.*), Ophiopogonis Radix (*Ophiopogon japonicus*), Astragali Radix (*Astragalus membranaceus* (*Fisch*.) *Bge. var. mongholicus* (*Bge*.)*Hsiao*), Schisandrae Chinensis Fructus (*Schisandra chinensis* (*Turcz*.) *Baill.*), Poria (*Poria cocos* (*Schw*.) *Wolf*), Rehmanniae Radix Praeparata (*Rehmannia glutinosa Libosch.*), Drynariae Rhizoma (*Drynaria fortunei* (*Kunze*) *J.Sm.*), and Chuanxiong Rhizoma (*Ligusticum chuanxiong Hort.*). All the herbs were provided by Beijing Tong Ren Tang Chinese Medicine Company, China, and met the criterion of *Pharmacopoeia of the People's Republic of China (the 2015 edition)*. The TKXLD formula was extracted under reflux with distilled water (1 : 10 volume) twice for 1 h each. After that, the extracts were concentrated to 100%, then filtered and dried below 60°C to obtain TKXLD granules. The TKXLD granule was dissolved in 70% solution using distilled water before use.

### 2.2. Animals and Treatments

40 male Wistar rats, weighing 170 g to 190 g (180 ± 10), were used in this study (Grade SPF/VAF, Certificate No: SCXK(Jing) 2002-2003, Beijing Laboratory Animal Research Center). All animal experiments were performed strictly in accordance with the guidelines of Beijing University of Chinese Medicine Animal Care and Use Committee. The animals were maintained at an ambient temperature of 16-20°C under a 12 h : 12 h light-dark cycle. Water and food were given ad libitum. The rats were randomly divided into four groups: the control group (C), tail-suspended group (S), tail-suspended adding irradiated group (SAI), and herb (Taikong Xieli Decoction, TKXLD) group, 10 rats per group.

Rats in the S, SAI, and TKXLD groups were subjected to tail suspended of head down position of -30° to simulate microgravity and those in the SAI and TKXLD groups were irradiated with 4.5 Gy of ^60^Co-gamma rays at the 8th day of tail suspended (the cobalt bomb was provided by Academy of military medical sciences). Rats of the TKXLD group were orally administrated with the decoction (7 g·kg^−1^·d^−1^) abstracted from a compound formula of traditional Chinese medicine and other groups with equivalent normal saline. All rats were anesthetized via an intraperitoneal injection of 2% sodium pentobarbital (0.25 mL/100 g) and then killed at 2 hours after intragastric administration, on the 21th day of the experiment. At that time, related tissues were extracted.

### 2.3. Proliferation of T Lymphocyte Measurement

Half of the spleen, which was extracted under aseptic condition, was put in a culture dish. 2 mL RPMI-1640 incomplete medium was added at the same time. After that, single-cell suspension was prepared by grinding and filtration with 80 mesh sieve. Single-cell suspension was seeded into 96-well plates at a density of 5 × 10^−6^ cells/mL and a volume of 100 *μ*L/well, ConA (Sigma, USA) was added into each well at a final concentration of 5 *μ*g/mL. The cells were incubated for 68 h at 37°C, 5%CO_2_ after 100 *μ*L solution was discarded in each well. Then, the culture media were eliminated and 10 *μ*L MTT solution (5 mL, Sigma, USA) was added into each well, followed by culturing at 37°C in a 5% CO_2_ humidified atmosphere for 4 h. 150 *μ*L DMSO (Sigma, USA) was then added and shook up. 10 minutes later, the absorbance of the solution was measured using a microplate reader at the wavelength of 570 nm.

### 2.4. Phagocytosis of Macrophage Measurement

The peritoneal fluid was obtained using cold Hank's with heparin washing. After that, the peritoneal macrophages were collected by draining the solution. The cell concentration was regulated to 5 × 10^−9^ cells/L and added into a 96-well plate which was put in the incubator. Four hours later, neutral red solution was added and then cell lysates were added after 40 minutes. Macrophage suspension was put over the night of 4°C; then, the absorbance of the solution was measured using a microplate reader at the wavelength of 492 nm.

### 2.5. Radioimmunoassay (RIA)

The hypothalamus was rapidly collected onto ice. After weighing, the hypothalamus was boiling in 1 mL normal saline for 3 minutes; 1 N glacial acetic acid (0.5 mL) was added and homogenized. The homogenate was neutralized with 1 N NaOH (0.5 mL) and centrifuged at 3000 r/min for 30 min. The supernatant was drained and stored at -80°C. The level of the corticotropin-releasing hormone (CRH) was assayed using a radioimmunoassay (RIA) kit (Haikerui Biotechnology Center, Beijing, China). Pituitary suspension was drained as mentioned above, and the level of adrenocorticotropic hormone (ATCH) was measured using a RIA kit (Huaying Institute of Biotechnology, Beijing, China). Furthermore, the levels of CRH and ACTH in plasma were, respectively, measured using a RIA kit as mentioned above. The level of CORT in the serum was measured using RIA kit (Huaying Institute of Biotechnology, Beijing, China). The supernatants of splenocyte suspension and macrophage suspension were, respectively, drained by measuring interleukin 2 (IL-2), interleukin 1 beta (IL-1*β*) and interleukin 6 (IL-6) using a RIA kit (Science and technology development center of PLA General Hospital, Beijing, China). IL-1*β*, IL-2, and IL-6 levels in the serum were, respectively, measured using the RIA kit as stated above.

### 2.6. Reverse Transcription-Polymerase Chain Reaction (RT-PCR)

Total RNA was extracted from the splenocytes using RNA TRIzol (Gibco, USA) and reverse transcription into cDNA. The primers for glucocorticoid receptor (GR) and *β*-actin were shown in Table 1. RT-PCR detected fluorescence, and the levels of mRNA were normalized to *β*-actin expression. Primers for RT-PCR were listed as follows.

### 2.7. Statistical Analysis

The experimental data was expressed as the mean ± standard deviation (*X̅*±SD). All the data were analyzed by one-way ANOVA using SPSS 23.0 software and differences were considered significant at *P* < 0.05.

## 3. Results

### 3.1. HPA Axis

#### 3.1.1. CRH

**Figure 1:**
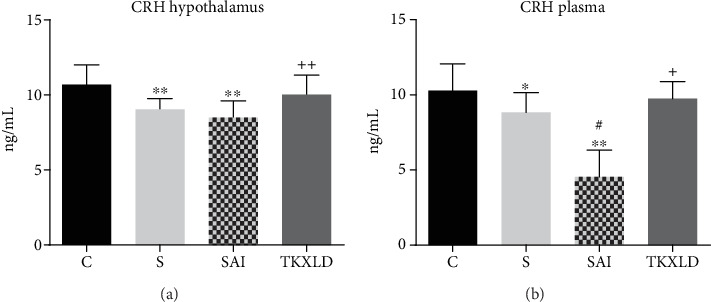
The level of CRH in the hypothalamus and plasma. (a) The level of hypothalamic CRH in each group. (b) The level of plasmatic CRH in each group. *N* = 10 for each group. ^∗^*P* < 0.05, ^∗∗^*P* < 0.01 compared with the control group. ^#^*P* < 0.05 compared with the S group. ^+^*P* < 0.05, ^++^*P* < 0.01 compared with the SAI group.

As shown in Figure 1, compared with the control group, the levels of the hypothalamic and plasmatic CRH in the S and SAI group were reduced significantly. Compared with the S group, the level of plasmatic CRH in the SAI group was reduced significantly. Compared with the SAI group, the levels of hypothalamic and plasmatic CRH in the TKXLD group was increased significantly.

#### 3.1.2. ACTH

**Figure 2:**
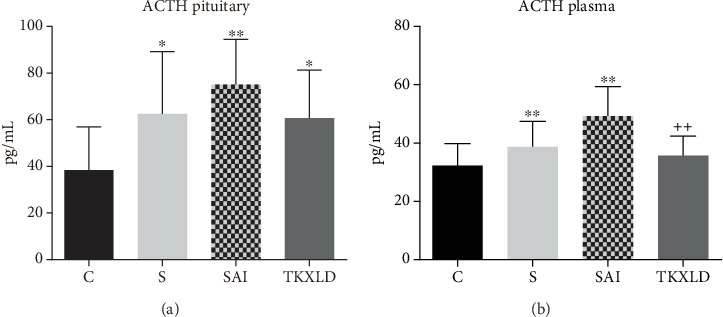
The level of ACTH in the pituitary and plasma. (a) The level of hypophyseal ACTH in each group. (b) The level of plasmatic ACTH in each group. *N* = 10 for each group. ^∗^*P* < 0.05, ^∗∗^*P* < 0.01 compared with the control group. ^++^*P* < 0.01 compared with the SAI group.

As shown in Figure 2, compared with the control group, the levels of pituitary and plasmatic ACTH in the S and SAI groups were both increased significantly. There was no statistical difference between the S and SAI groups. Compared with the SAI group, the levels of pituitary and plasmatic ACTH in the TKXLD group were reduced significantly.

#### 3.1.3. CORT and GR mRNA

**Figure 3:**
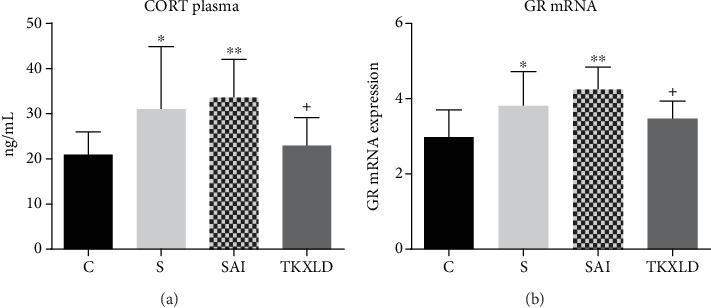
The expression of plasmatic CORT and splenic GR mRNA. (a) The level of plasmatic CORT in each group. (b) The expression of splenic GR mRNA in each group. *N* = 10 for each group. ^∗^*P* < 0.05, ^∗∗^*P* < 0.01 compared with the control group. ^+^*P* < 0.05 compared with the SAI group.

As shown in Figure 3(a), compared with the control group, the levels of plasmatic CORT in the S and SAI groups were both increased significantly. For CORT, there was no significant difference between the S and SAI groups. Compared with the SAI group, the level of CORT in the TKXLD group was reduced significantly. As shown in Figure 3(b), compared with the control group, the expression of splenic GR mRNA in the S and SAI group were both increased significantly. There was no significant difference in the GR mRNA expression between the S and SAI groups. Compared with the SAI group, the GR mRNA expression in the TKXLD group was reduced significantly.

### 3.2. Immune Function

#### 3.2.1. T-Cell Function and Related Cytokines

**Figure 4:**
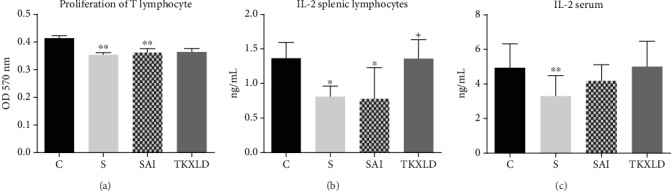
T-cell proliferation and related IL-2 level. (a) The proliferation of splenic T-cell in each group. (b) The level of splenic IL-2 in each group. (c) The level of serous IL-2 in each group. *N* = 10 for each group. ^∗^*P* < 0.05, ^∗∗^*P* < 0.01 compared with the control group. ^+^*P* < 0.05, ^++^*P* < 0.01 compared with the SAI group.

As shown in Figures 4(a) and 4(b), compared with the control group, the capacity of splenic T-cell proliferation and IL-2 secreting capacity in the S and SAI groups were decreased significantly. There was no significant difference of splenic T-cell proliferation and IL-2 secreting capacity between the S and SAI groups. Compared with the SAI group, the IL-2 secreting capacity in the TKXLD group was increased significantly. As shown in Figure 4(c), compared with the control group, the level of serous IL-2 in the S and SAI groups was decreased significantly. There was no statistical difference of serous IL-2 between the S and SAI groups. Compared with the SAI group, the levels of serous IL-2 in the TKXLD group had an uptrend, but there was no significant difference.

#### 3.2.2. Celiac Macrophage Function and Related Cytokines

**Figure 5:**
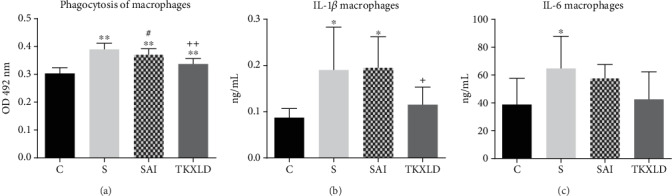
Macrophagic phagocytosis and related cytokine levels. (a) Phagocytosis of celiac macrophages in each group. (b) The level of macrophagic IL-1*β* in each group. (c) The level of macrophagic IL-6 in each group. ^∗^*P* < 0.05, ^∗∗^*P* < 0.01 compared with the control group. ^#^*P* < 0.05, ^##^*P* < 0.01 compared with the S group. ^+^*P* < 0.05, ^++^*P* < 0.01 compared with the SAI group.

As shown in Figure 5, for celiac macrophages, the phagocytosis and IL-1*β* level in the S and SAI groups were both significantly enhanced in comparison with the control group. The macrophagic IL-6 level in the S group was significantly increased in comparison with the control group. Compared with the S group, the phagocytosis of macrophages in the SAI group was decreased markedly, and the levels of IL-1*β* and IL-6 showed no statistical difference. Compared with the SAI group, the phagocytosis and IL-1*β* level of macrophages in the TKXLD group was decreased significantly.

**Figure 6:**
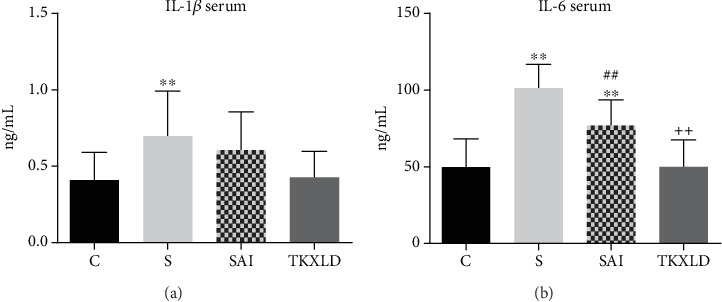
Serous IL-1*β* and IL-6 levels. (a) The level of serous IL-1*β* in each group. (b) The level of serous IL-6 in each group. *N* = 10 for each group. ^∗^*P* < 0.05, ^∗∗^*P* < 0.01 compared with the control group. ^#^*P* < 0.05, ^##^*P* < 0.01 compared with the S group. ^+^*P* < 0.05, ^++^*P* < 0.01 compared with the SAI group.

As shown in Figure 6, compared with the control group, the levels of serous IL-1*β* and IL-6 in the S and SAI groups were significantly increased. There was no significant difference of serous IL-1*β* between the S and SAI groups, but the level of serous IL-6 in the SAI group was significantly decreased compared with the S group. There was no significant difference of serous IL-1*β* between the SAI and TKXLD groups. However, the level of serous IL-6 in the TKXLD group was significantly decreased compared with the SAI group.

## 4. Discussion

Microgravity and irradiation are two main factors affecting organisms during spaceflight. In this study, we simulated the microgravity state of a rat by tail suspension. Meanwhile, we also compared the neuro-endocrine-immune influence under microgravity-irradiation environment with microgravity state. Recently, there is a wide range of chosen irradiation dosage which is from 0.5 Gy to 25 Gy on space irradiation research [12–14]. The designed irradiation dose is determined by species, experimental purpose, and biological endpoints [15]. Considering that the astronauts might suffer high-dose space irradiation during solar nucleon active period or extravehicular activities, and the Wistar rat had a relative low sensitivity in comparison with other experimental animals, we used a total of 4.5 Gy irradiation dosage (0.3 Gy/min) which was a little bit higher than the commonly used irradiation dosage (2.5 Gy or thereabouts). In this article, we found that, compared with the control group, the level of CRH was decreased, the ACTH and CORT level were increased, and the expression of splenic GR mRNA was upregulated in both the S and SAI groups. The proliferative and IL-2 secreting capacity of T-cell in the S and SAI group were weakened, but the phagocytosis and related cytokine secreting capacity of celiac macrophages were significantly increased. On the other hand, the plasmatic CRH, phagocytosis of macrophages, and serous IL-6 in the SAI group were all decreased significantly compared with the S group. Furthermore, TKXLD could normalize the anomalous changing of the NEI targets to varying degrees.

There is a close correspondence between the neuroendocrine and immune systems, both of these two systems constitute the complex NEI system. As an important part of the NEI system, there also exists a two-way communication and feedback between the HPA axis and the immune system. For one thing, plenty of cytokines like IL-1, IL-6, IL-10, and tumor necrosis factor alpha (TNF-*α*) could activate the HPA axis; for another, HPA could regulate the systemic inflammatory response. HPA axis is activated under hyperinflammatory state; high-level glucocorticoids are then released to inhibit the immune response by hindering the proinflammatory cytokines secreting like IL-1 and TNF-*α*. Meanwhile, the HPA axis could induce immune cells releasing anti-inflammatory cytokines such as IL-4, IL-10, and IL-13 to restrain the inflammation, which contributes to protecting the body from the damages caused by the excessive activation of the immune system [16].

In the microgravity state, the change of gravity leads to the imbalance of body fluids. The biofluid flows to the head, which causes the change of cerebral blood flow and hemodynamics. The cerebral metabolism, including the central nervous system (CNS) metabolism, is then adversely affected. The expression of hypothalamic proteins is disordered including oxidative imbalance [17]. The feeding frequency of the pituitary cells was disrupted, which leads to hormone secretion disorder [18]. Furthermore, the levels of humoral adrenaline, noradrenalin, dopamine, ACTH, growth hormone (GH), prolactin, and CORT were increased at the same time [19]. The redistribution of body fluids could also induce the stress response of the immune system. In this state, for immune organ atrophy, the T-cell and B-cell secreting capacity of which falls into decline simultaneously [20]. Additionally, the expression and ability of maturation markers on dendritic cells (DC) were dwindled [21]. The resistance of the body to some pathogens is enhanced by activating macrophages [22].

Irradiation could act upon the external body parts such as the skin or retina directly and influence the internal system or tissues via the bystander effect, which is different from microgravity. It is reported that radiotherapeutic irradiation could lead to endocrine dyscrasia, pituitary insufficiency, and HP axis dysfunction [23]. Space irradiation is reported to injure the hypothalamus, prefrontal lobe, and nucleus accumbens [24]. It is also reported that space irradiation could give a promotion on macrophage proliferation and enhance its phagocytic function [25].

Organisms are threatened with microgravity, irradiation, and other extreme environmental hazards in the space environment. The NEI system shows the approximate changes under space environment, including the increasing ACTH, thyroxine, CORT, and antidiuretic hormone (ADH) levels [26]; the hypofunction on T-cell [27]; and overactivation on macrophages [28].

The influence of microgravity and irradiation on the human body nearly has the similar trend, but each of which is emphasized in different directions further. It is reported that the human body could be caused to lower the CNS defense against oxidative damage [29] and reduce the quantity of lymphocytes [30] under either microgravity or irradiation condition. Remarkably, it is reported that space flight-associated anorexia and musculoskeletal degenerative changes may be driven by irradiation- and microgravity-associated mechanisms, respectively [31], which suggested that there existed a different intervention mechanism, respectively, between microgravity and irradiation. A raft of research suggested that there might be a kind of “superposition effect” on the organism while it was under the condition of both microgravity and irradiation. It is reported that the apoptosis rate of the Hmy2.CIR cell, which is the metrocyte of B-cell, under the conditions of microgravity-irradiation was way above which under the condition of single irradiation [32]. Microgravity could aggravate the genotoxicity caused by irradiation [33]. Compared with microgravity, the microgravity-irradiation environment showed the most serious oxidative stress in the cerebral cortex of mice [34].

In this study, we found that the HPA axes and macrophages in both the S group and SAI group were unanimously active, but the splenic T-cell and related cytokines were under hypofunctional state. Compared with the S group, the level of plasmatic CRH, macrophage activity, and serous IL-6 were all decreased significantly, which indicated that irradiation could antagonize the HPA and macrophage stress caused by microgravity, as well as aggravate the hypofunction of T-cell. Upon this, we hypothesized that there might be several differences on the body stress mechanism between microgravity and microgravity-irradiation, while irradiation might weaken the self-adaptive ability under the condition of microgravity.

The HPA axis consists of the hypothalamus, pituitary, and adrenal glands. CRH and arginine vasopressin (AVP) which are synthesized in the relay neurons of the paraventricular hypothalamic nucleus (PVN) are released into the anterior pituitary via the portal vein. ACTH is driven to release after that, which could activate adrenocortical cells to synthesize and secrete CORT and GC that contributes to energy mobilization and homeostasis maintaining via negative feedback regulation [35]. In this research, the ATCH and CORT levels in the S and SAI groups were both elevated, which illustrated that the HPA axis was activated. As the level of CRH was observed to be significantly decreased in the S and SAI groups, it might be caused by the hypothalamic inverse feedback signal that was given by the overexpressed GR and glucocorticoid which was caused by systemic stress.

There is a close correspondence between the HPA axis and the immune system. ACTH is known as an important immune modulator that could regulate the phagocytosis of macrophages [36]. On the one hand, a high level of CORT could lead to the inhibition of immunity and inflammation. On the other hand, the acute release of CORT could also upregulate the expression of IFN-*γ* receptor and promote macrophages secreting IL-6 [37]. The plasmatic CRH, mainly coming from epithelial cells and immunocyte secretion, besides hypothalamic transportation through the axons of nerve endings, could regulate cytokines via autocrine and paracrine [38]. Compared with T-cell or B-cell, macrophage shows the most sensitive immune activity to CRH [39]. CRH is indicated to promote the phagocytosis of macrophages via the PKA/PKC-ERK1/2-RhoA/Rac1 signaling pathway [40]. In this study, we indicated that the overexpression of CORT and GR mRNA led to the inhibition of T-cell function and enhancement of macrophage activation. Microgravity combined with irradiation could downregulate the plasmatic CRH level to inhibit the hyperfunction of macrophages to some extent.

TKXLD is composed of Ginseng, Ophiopogonis, Radix Astragali, Schisandrae Chinensis Fructus, Poria, Rehmanniae, Drynariae, and Chuanxiong. In TCM theory, TKXLD is able to replenish Qi, nourish Yin, toxify the kidney, and promote blood circulation. It is reported that Ginseng and Radix Astragali show favorable antimicrogravity [41, 42] and anti-irradiation effects [43, 44] that could regulate immune response and HPA axis dysfunction caused by stress to maintain the NEI balance [45, 46]. Ophiopogonis Radix is proved to contribute to ameliorate macrophage activity [47]. There are evidences that Schisandrae Chinensis Fructus and Chuanxiong have protective effects on irradiation damage, the mechanisms of which contains reversing damage caused by irradiation and inhibiting the overactivation of the HPA axis [48–50]. Poria has been used as a good immune modulator that could resist the excessive macrophage activation induced by LPS [51]. Rehmanniae has been proved to be efficacious in retaining NEI homeostasis [52]. Drynariae is considered a kind of potential antimicrogravity medicine that has a good effect on several diseases like bone loss led by microgravity [53]. Thus, it is indicated that the compatibility of the herbs above might produce a marked effect on NEI imbalance caused by irradiation and microgravity. In this research, for the rats in the TKXLD group, the amelioration of the HPA axis, macrophage, and splenic T-cell function was observed after TKXLD treatment. Meanwhile, the expression of splenic GC mRNA was downregulated in the TKXLD group. All these evidences have demonstrated that TKXLD has a protective influence on the NEI system of rats under microgravity-irradiation conditions. The mechanism of TKXLD might be involved in different function links of CNS and peripheral parts.

Human body faces dual challenges of microgravity and irradiation during spaceflight. Current studies on physiological adaptation in space are mostly focused on the single environmental factors. There is an urgent need for physiological research that is studied under complex environments. In this article, we entirely compared the functional changes of the NEI system between microgravity and microgravity-irradiation. The efficacy of TKXLD on treating NEI dysfunction caused by microgravity-irradiation was also investigated. The results indicated that the rats were suffered from dysfunction of the NEI system in different degrees under both microgravity and microgravity-irradiation, but there still are some differences, especially those related to the pathogenesis between the two conditions. Our study might be conducive to exploring the physiological adaptation mechanism of the human body in space, developing the defense strategy against extreme space environment, and promoting the protective use of TCM on spaceflight.

However, we only observe the NEI change in medium-term spaceflight of 21 days for the rats. Only the HPA axis and representative immunocytes in the NEI system were investigated. Though we found that irradiation might superimpose its effect on microgravity by messing the NEI system, the bioregulating mechanism against microgravity-irradiation among the subsystems of the NEI network still deserved to be explored in depth.

## 5. Conclusion

Both microgravity and microgravity combined with irradiation could lead to the dysfunction of the HPA axis and immunity. The hyperfunctional HPA axis might cause dysimmunity. Irradiation could not only aggravate the negative feedback inhibition of the HPA axis but also antagonize the macrophage hyperfunction caused by microgravity. TKXLD could favorably ameliorate the dysfunction of the NEI system caused by microgravity-irradiation.

## Figures and Tables

**Table 1:** Primers for RT-PCR.

Gene	Primer sequence (5′→3′)	Product size (bp)
GR	Forward:ACCCTGCTACAGTACTCATGGA Reverse:CTTGGCTCTTCAGACCTTCCT	271
*β*-Actin	Forward:CATCCTGCGTCTGGACCT Reverse:CACACAGAGTACTTGCGCTCA	498

## Data Availability

The data used to support the findings of this study are available from the corresponding author upon request.
